# Synthetic Genetic Elements, Devices, and Systems

**DOI:** 10.3390/life12070945

**Published:** 2022-06-23

**Authors:** Yusuke Kato, Chunbo Lou

**Affiliations:** 1 Institute of Agrobiological Sciences, National Agriculture and Food Research Organization (NARO), Oowashi 1-2, Tsukuba 305-8634, Ibaraki, Japan; 2 Center for Cell and Gene Circuit Design, CAS Key Laboratory of Quantitative Engineering Biology, Guangdong Provincial Key Laboratory of Synthetic Genomics, Shenzhen Institute of Synthetic Biology, Shenzhen Institutes of Advanced Technology, Chinese Academy of Sciences, Shenzhen 518055, China

Since the beginning of life on Earth, over the course of 3 to 4 billion years, nature has created vast quantities of genetic elements. These freshly created elements faced natural selection: winners survived, and losers disappeared. Nature selected only the genetically stable elements that contributed to the maintenance of life. From a contrary perspective, natural selection restricted the diversity of genetic elements. In the last 20 years, i.e., 2 × 10^−8^ billion years, synthetic biologists have tried to create novel genetic elements that nature has not invented or cannot invent. The objective of this research is to go beyond the restriction of natural selection and obtain novel genetic elements that are useful for human use. From the limited modification of the characteristics of natural elements to originally designed elements, various synthetic genetic elements have been reported. “Genetic devices”, such as logic gates and memory elements, and higher order “genetic systems”, such as metabolite factories and biological containment systems, can be constructed using these synthetic elements in combination with other genetic elements. Through this approach, the incorporation of synthetic genetic elements is dramatically expanding biological functions.

The Special Issue “Synthetic Genetic Elements, Devices, and Systems” published in *Life* (ISSN 2075-1729) collects a series of research and review articles related to synthetic genetic elements, circuits as devices, and systems. This issue contains 11 articles. The following is an overview.

## 1. Synthetic Genetic Switches

Five of the papers focus on one of the most important techniques in synthetic biology: the process of controlling the expression of specific genes.

Transcriptional switches are the most fundamental and important genetic elements for building synthetic gene circuits. Transcription switches have been developed mostly in prokaryotes, but in recent years there have also been remarkable developments in eukaryotes. Tominaga et al. present a comprehensive review of synthetic genetic switches working at the transcriptional level in yeast [1]. In particular, this review provides detailed information on how to improve these switches, including the latest directed evolution procedures.

Functional noncoding RNAs, such as riboswitches, ribozymes, and aptamers, are useful components of synthetic genetic circuits. Riboswitches regulate transcription, translation, RNA splicing, and RNA stability, while ribozymes have catalytic activity similar to that of protein enzymes. Aptamers interact with specific molecules, and riboswitches and ribozymes can be fused with aptamers to express their functions in a conditional manner. Ge and Marchisio present a comprehensive review of the aptamers, riboswitches, and ribozymes used in yeast synthetic biology [2].

The development of modular, orthogonal, and easily programmable genetic components to build more complex and sophisticated synthetic genetic circuits is highly desirable. Clustered Regularly Interspaced Short Palindromic Repeats (CRISPR) systems can be used as a genetic switch with these characteristics, as well as for genome editing. Du et al. present a comprehensive review of CRISPR-based genetic switches and their applications, including in bio-computation [3].

The toehold switch sensor is a riboregulator that controls the translation of downstream genes. The addition of a trigger RNA initiates translation by releasing the RBS and the translation initiation codon by changing the secondary structure of the toehold switch sensor. Incorporating a toehold switch sensor into the expression construct of a reporter gene allows for the detection of RNAs of interest, such as viral RNAs. However, when the trigger RNA is too long, the formation of secondary structures often makes detection problematic. Heo et al. report that the use of helper RNAs that inhibit the secondary structure formation in a trigger RNA is effective for the detection of *E. coli pks* island mRNAs, which have been linked to colorectal cancer [4].

The toggle switch is an important genetic device that plays a central role in both natural and synthetic gene systems. However, it is difficult to ensure predictable system-level behaviors because toggle switches display a particularly strong dependence on the genetic context. Gyorgy reports an extensive analysis of the genetic toggle switch in the presence of leaky promoters and the cellular burden using a mathematical model [5].

## 2. Protein-Based Regulatory Systems

DNA transfection is the standard method for transgene expression, but the risk of insertional mutations is a drawback for clinical use, such as cancer therapy. The direct administration of synthesized mRNA can avoid the risk of insertional mutations. However, for spatiotemporal and cell-specific control, novel methods distinct from DNA transfection are required. Nakanishi reviews protein-based translational regulatory systems for synthetic mRNAs in mammalian cells [6]. It is noteworthy that the synthetic mRNAs can express both effector proteins and regulatory proteins, allowing for the construction of multilayered genetic circuits composed entirely of mRNAs.

Reversible protein–protein interactions, such as those involving binding to allosteric regulators, structural changes in upstream regulators, and post-translational modifications, respond within seconds and are much faster than mechanisms that occur via transcription or translation. Rosa et al. review synthetic protein circuits and devices based on such reversible protein–protein interactions [7].

## 3. Application to Cell Factories

The production of chemicals and proteins in cell factories is one of the most important applications of synthetic genetic systems. The last four articles address this research field.

Techniques for the incorporation of unnatural amino acids into ribosomally synthesized proteins have been developed in the recent decades. The amber stop codon has usually been selected for unnatural amino acid incorporation. Kato proposes a novel method to control the growth and viability of target bacteria by incorporating unnatural amino acids at sense codons, causing the production of non-functional or toxic proteins [8]. This method is promising as a means of regulating the growth of microorganisms used in cell factories, especially in controlling the proper proportion of mixed populations.

Human embryonic kidney 293 (HEK293) cells are a human-derived cell line that is commonly used to produce therapeutic glycoproteins. Mild hypothermia has been widely used to enhance transgene expression in HEK293 cells. Jang, Min, and Lee report promoter-dependent hypothermia responses in HEK293 cells that can be attributed to the interaction of exogenous promoters with endogenous transcription factors [9]. These findings could facilitate the construction of industry-relevant mild-hypothermia-inducible promoter hybrids.

Uridine diphosphate-glucose dehydrogenase (UGD) is an enzyme that produces uridine diphosphate-glucuronic acid, a precursor for useful glycosaminoglycans such as hyaluronic acid, heparosan, and chondroitin. The step catalyzed by UGD is considered to be the limiting factor in the heterologous production of glucosaminoglycans. Couto et al. have characterized three new UGDs and propose new enzyme options for industrial use [10].

In synthetic biology, gene clusters are reconstituted using controllable genetic elements to optimize the production of desired metabolites. Towards this end, transcriptional regulation using well-characterized promoters is an important approach. For metabolic pathways involving multiple operons, adjusting each promoter strength is important to obtaining maximum production efficiency. Zhao et al. report a markerless and combinatorial approach to obtaining an optimal balance of multiple promoters controlling individual operons, resulting in the improved production efficiency of thaxtomin A [11].

We hope that this collection will help researchers to develop novel synthetic genetic elements, circuits, devices, and systems.
